# Prospective study on the Eustachian tube function during Frenzel maneuver in a hypobaric/hyperbaric pressure chamber

**DOI:** 10.1007/s00405-021-06888-1

**Published:** 2021-06-06

**Authors:** Philipp Wolber, Moritz Friedo Meyer, Kristijana Knesic, Svenja Rink, Stefanie Jansen, Jens Peter Klussmann, Maria Grosheva

**Affiliations:** 1 Department of Otorhinolaryngology, Head and Neck Surgery, University of Cologne, Kerpener Str. 62, 50924 Cologne, Germany; 2 Department of Otorhinolaryngology, Head and Neck Surgery, University Duisburg-Essen, Essen, Germany; 3 Department of Prosthetic Dentistry, School of Dental and Oral Medicine, University of Cologne, Cologne, Germany

**Keywords:** Middle ear ventilation, Eustachian tube, Valsalva maneuver, Diving, Pressure equalizing maneuvers

## Abstract

**Introduction:**

The Frenzel maneuver describes a technique for middle ear equalizing which is frequently used by apnea divers. It offers advantages compared to the most commonly used techniques such as the Valsalva or Toynbee maneuver. Until now, there is insufficient literature about the pressure dynamics and Eustachian tube (ET) function during the Frenzel maneuver. The aim of the present study was to characterize the ET function during the Frenzel maneuver.

**Materials and methods:**

By means of an established standardized profile of compression and decompression in a hypo/hyperbaric pressure chamber, we examined different parameters such as the ET opening pressure (ETOP), ET opening duration (ETOD), and ET opening frequency (ETOF) in 11 experienced apnea divers and compared them to the parameters during the Valsalva and Toynbee maneuver.

**Results:**

Standard values for ETOP, ETOD, and ETOF could be established for the Frenzel maneuver under standardized conditions in a hypo/hyperbaric pressure chamber. Compared to the Frenzel maneuver, ETOP was higher and ETOD longer (both *p* < 0.001) during the Valsalva maneuver whereas ETOP was lower and ETOD shorter (both *p* < 0.001) during the Toynbee maneuver. No difference regarding ETOF was observed between the Frenzel, Valsalva, and Toynbee maneuver.

**Discussion:**

The Frenzel maneuver was shown to be at least as effective as the Valsalva maneuver concerning ET opening. We believe that knowledge of the Frenzel technique might facilitate the pressure equalization during diving and recommend implementation of an appropriate equalization training in apnea and scuba diving education.

**Supplementary Information:**

The online version contains supplementary material available at 10.1007/s00405-021-06888-1.

## Introduction

To descend to depths quickly, apnea divers must be capable of equalizing middle ear pressure effortlessly and efficiently [1]. The Frenzel maneuver is often named as the most popular equalizing technique. In apnoeist circles, it is speculated that this method is the fastest and easiest way to equalize pressure. There are several maneuvers for pressure equalization in the middle ear and (naso) pharynx. The most commonly used technique is the Valsalva maneuver. This maneuver is performed by moderately forceful attempted exhalation against a closed airway, usually performed by closing one’s mouth and pinching one’s nose shut while expelling air out as if blowing up a balloon. This technique, which is primarily aimed to open the Eustachian tube (ET), was first described by Antonio Maria Valsalva in the seventeenth century in Bologna [2]. However, besides the need for a helping hand for closure of the nose, the Valsalva maneuver might be associated with complications, as rupture of the round or oval window in the middle ear in case of over-pressurization. It can also lead to increased intrathoracic pressure resulting in changed cardiac hemodynamics and obstructed venous return. In case of a persisting foramen ovale, use of the Valsalva technique might cause a significant right–left shunt in the atrium [2]. Another commonly used technique is the Toynbee maneuver. It can be performed by simple swallowing and thereby compressing air against the ET by moving the tongue [3].

The Frenzel maneuver was first described in 1938 and is named after the German ENT-specialist Hermann Frenzel. The maneuver was originally taught to military personnel during The Second World War [4, 5]. Its execution involves voluntary closure of the glottis, mouth, and nose and subsequent contraction of the muscles of the floor of the mouth and superior pharyngeal constrictor muscles. This enables compression of air in the nasopharynx and up both ET. In simple terms, one tries to equalize the pressure with a closed mouth and nose and producing the sound of the letter “K”. The maneuver can be performed hands-free using a nose-clip and at any level of inspiration [2, 6]. The effort to perform the Frenzel maneuver is minimal, and it can be repeated many times very quickly. However, it is not to be confused with the true hands-free Delonca or BTV (béance tubaire volontaire) technique. BTV is defined as a voluntary opening of the ET and is named after Georges Delonca. This method requires voluntary control of tensor veli palatine muscles, which leads to opening of the ET [7].

While there are many studies about the characterization and function of the Valsalva and Toynbee maneuvers, there is insufficient literature about the pressure dynamics and ET function during the Frenzel maneuver. Moreover, no standardized definition of the Frenzel maneuver has been reported so far. Pressure chamber-based studies enable to perform reproducible measurements of the ET function under standardized conditions. Therefore such studies pose an objective and reliable method to characterize the Frenzel maneuver for the first time [8]. By means of an established standardized profile of compression and decompression in a hypo/hyperbaric pressure chamber different parameters such as the ET opening pressure (ETOP), ET opening duration (ETOD), and ET opening frequency (ETOF) can be evaluated and describe the dynamic function of the Eustachian tube [9].

The aim of the present study was to characterize the ET function parameters during the Frenzel maneuver in experienced apnea divers under standardized conditions in a hypo/hyperbaric pressure chamber and compare it to the parameters during the Valsalva and Toynbee techniques.

## Materials and methods

### Ethical considerations

This design and the protocol of this study was approved by the Ethics Committee of the University of Cologne (local register number 18–427) and was registered in the German Clinical Trials Register (No. DRKS00017289). The study was performed according to the Declaration of Helsinki. All participants signed a consent form before participation.

### Inclusion criteria

We included 11 [11] healthy apnea divers. None of the participants had been (free) diving 24 h prior to the study. Everybody was familiar with and able to execute the Frenzel maneuver.

### Pressure chamber profile

**Fig. 1:**
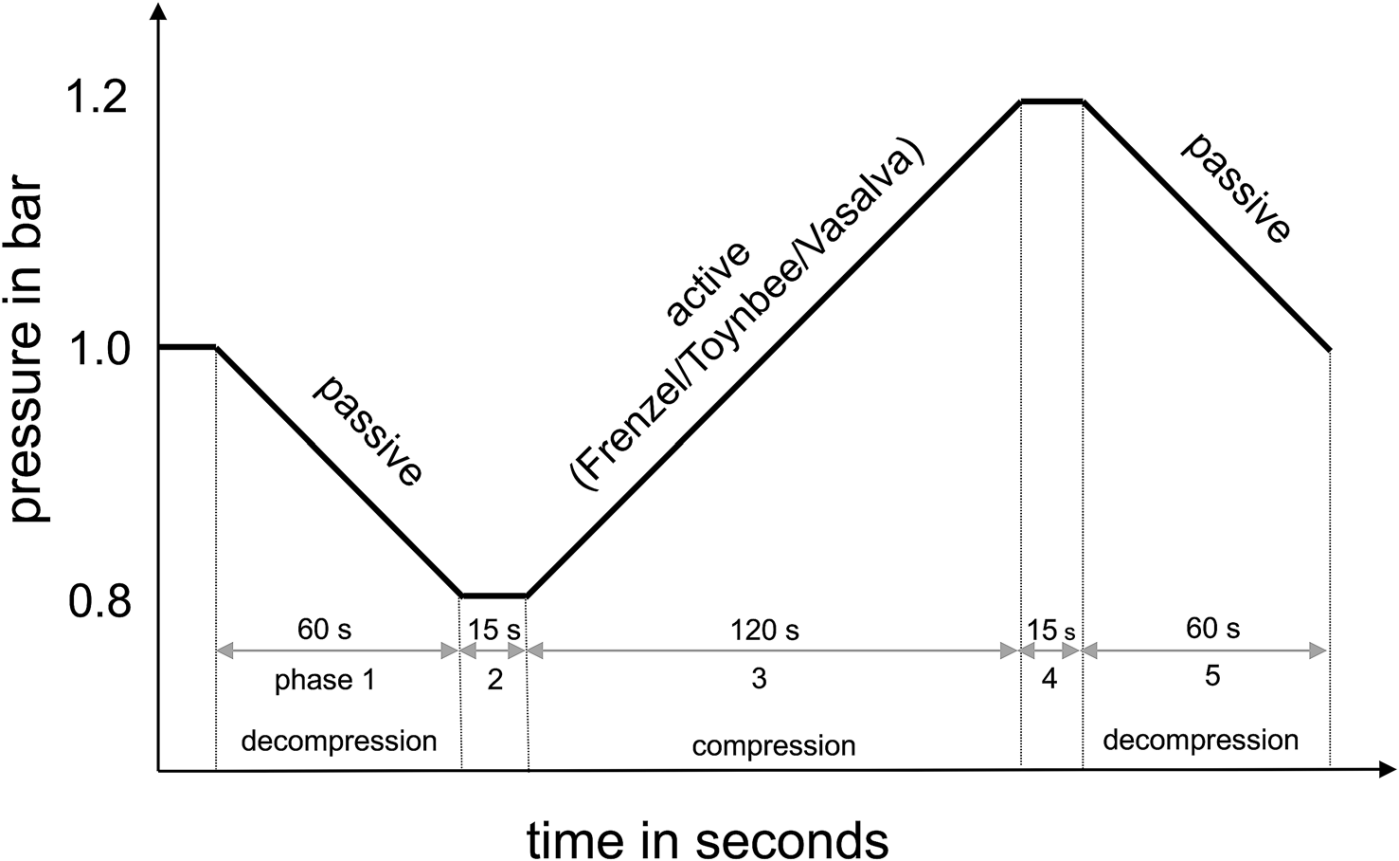
Standardized hypobaric/hyperbaric pressure chamber profile of compression and decompression start at 1.0 bar with a decompression over 60 s–0.8 bar (phase one is passive pressure equalization), remaining at 0.8 bar in isopression for 15 s (phase two), compression to1.2 bar over 120 s (phase three is active pressure equalization phase by means of Frenzel maneuver (first measurement), Valsalva or Toynbee maneuver (second measurement), remaining at 1.2 bar in isopression for 15 s (phase four), decompression to 1.0 bar over 60 s (phase five). The pressure changes were performed with a steady speed of 0.2 bar per minute

Measurements were conducted in a single-person chamber (Haux Life Support, Karlsbad, Germany) with a standardized profile of compression and decompression as described earlier (Fig. 1 and supplementary Fig. 1) [8, 9]. Continuous impedance measurements (tympanometry) were performed via an ear probe containing a miniature loudspeaker, microphone and a small tube to allow pressure equalization between the ear canal and the chamber pressure environment. A continuous 226 hz probe tone was delivered during measurements and sound pressure level in the ear canal was evaluated reflecting impedance of the tympanic membrane. The pressure chamber profile consisted of five phases (Fig. 1).

Two separate pressure chamber measurements were carried out for this study. Between the two measurements, participants left the pressure chamber for approximately 15 min. During phases of decompression, no actively induced pressure equalization was allowed and passive openings of the ET were observed (Fig. 1, phase one). During the compression phase of the first measurement, pressure equalization was performed using the Frenzel maneuver with one hand. During compression phase of the second measurement, participants were asked to perform active middle ear pressure equalization either by swallowing (Toynbee maneuver) or by the Valsalva maneuver (Fig. 1, phase three).

### Clinical examination

Prior to each pressure chamber measurement, an otoscopic examination was carried out to exclude pre-existing pathologies in the external ear canal or in the middle ear and to exclude a significant barotrauma. Otoscopic findings were classified using the TEED classification, for the right and the left ear separately [10, 11]. TEED level 0 defined a normal tympanic membrane, TEED 1 a retraction and increased vascularisation of manubrium and shrapnel’s membrane, TEED 2 a retraction and hyperaemia of the entire tympanic membrane, TEED 3 fluid or blood in the middle ear, and TEED 4 perforated tympanic membrane.

### Eustachian tube function parameter

The following parameters of the ET function were analyzed from the software acquired data (Tube function exe) ET opening pressure (ETOP), ET opening frequency (ETOF), and ET opening duration (ETOD). ETOP in mbar defined the pressure in the middle ear at which the patient had actively started pressure equalization. ETOF indicates the number of ET openings per minute. ETOD defines the time span between opening and closing of the ET. Evaluation of the ET parameters in detail are demonstrated in previously published articles [8, 9, 12, 13].

### Statistical evaluation

The data for the left and right ear were collected separately and analyzed together (11 participants with 22 ears). The pressure chamber compression/decompression profile run automatically. The data on ET function parameters were continuously measured and displayed by the tube function.exe software. The parameters ETOP, ETOF, and ETOD were analyzed manually for each participant after each measurement separately.

All data were pseudonymized for statistical analysis. Statistical analysis was carried out using SPSS software (IBM SPSS Statistics version 26.0, IBM, New York City, NW, USA). ETOP, ETOF and ETOD during the Frenzel maneuver were characterized and then compared to the Valsalva and the Toynbee maneuver respectively. Because of abnormally distributed data, we used the Wilcoxon Signed rank test and Kruskal–Wallis test, respectively, to compare quantitative data of the independent groups. A *p* value of less than 0.05 was considered statistically significant, even though not corrected for multiple testing. All reported *p* values are two-sided.

## Results

### Participants’ characteristics

All of the included 11 participants were active freedivers, three of them women. Mean age was 52.6 ± 8.4 years. The participants were diving for an average time of 8.8 ± 6.2 years and completed on average 114.5 ± 99.6 freediving sessions with maximum dive depth of 30.3 ± 8.7 m. The last dive was carried out 2.1 ± 3.5 month ago, on average. During the first pressure chamber run, all participants [11] were told to equalize using the Frenzel maneuver. During the second pressure chamber measurement, five [5] participants performed the Toynbee maneuver and six [6] the Valsalva maneuver for active pressure equalization.

### Otoscopic findings

**Fig. 2:**
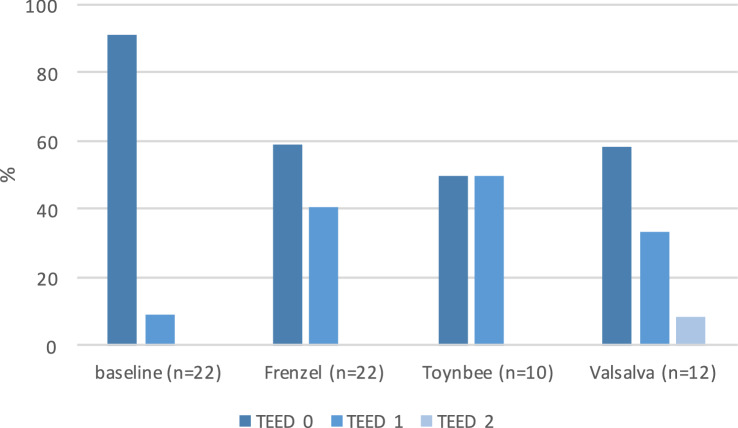
Otoscopic findings classified by TEED level Otoscopy was performed at baseline before measurement, after the first measurement using the Frenzel maneuver and after the second measurement using either the Toynbee maneuver or the Valsalva maneuver. TEED 0 is normal tympanic membrane TEED 1 is retraction and increased vascularization of manubrium and shrapnel’s membrane; TEED 2 is retraction and hyperaemia of the entire tympanic membrane

Before pressure chamber measurements, 90.9% were characterized as TEED 0 and 9.1% as TEED 1. After the first pressure chamber measurement using the Frenzel technique, TEED 0 was present in 59.1% and TEED 1 in 40.9% of the ears. After the second pressure chamber run (Valsalva or Toynbee), 54.5% of the ear findings were normal (TEED 0). In 40.9% of the ears, otoscopy revealed changes to TEED 1 and in 4.5% to TEED 2. After the Valsalva maneuver, TEED 0 was present in 58.3%, TEED 1 in 33.3% and TEED 2 8.3%. Of note, the one participant with TEED 2 showed a failed pressure equalization using the Valsalva maneuver and noted a slight hearing impairment during the measurement. After the Toynbee maneuver, TEED 0 and TEED 1 were each present in 50%, no TEED 2 was found. In total, no otoscopy changes with TEED level 3 or 4 were present (Fig. 2). The number of participants with TEED > 0 during otoscopy increased with cumulative pressure exposure, though not significantly (*p* = 0.085, Person’s chi-squared test, Fig. 2).

### Impedance curves of Frenzel, Valsalva, and Toynbee maneuvers

**Fig. 3:**
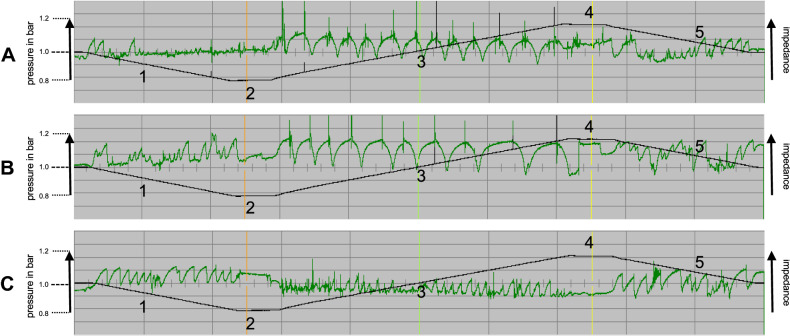
Typical impedance curve for one ear. The figure demonstrates the standardized pressure chamber profile during all phases 1–5 (for details see Fig. 1) with active pressure equalization (phase three) for the Frenzel (**A**), Valsalva (**B**) and Toynbee maneuver (**C**)

The typical impedance curves for Frenzel, Valsalva, and Toynbee maneuvers during actively induced pressure equalization in the compression phase (phase three) are shown in Fig. 3.

### Standard values for Frenzel maneuver

During performance of the Frenzel maneuver, ETOP for all 22 ears was 22.7 ± 33.7 mbar (range 2–328 mbar, 95% CI 18.6–26.8) with a median of 13 mbar. Correspondingly, ETOD was 2 ± 1.6 s (range 0.3–10.1 s, 95% CI 1.8–2.2) with a median of 1.8 s. ETOF was 5.9 ± 5 (range 0–17.5, 95% CI 3.7–8.1) openings per minute with a median of 4.5 times. ETOP, ETOF, and ETOD values for the Frenzel maneuver of all participants are shown in Table 1.

**Table 1:** Eustachian tube parameters for all participants during the Frenzel maneuver Eustachian tube opening pressure (ETOP, in mbar), Eustachian tube opening duration (ETOD, in seconds), and Eustachian tube opening frequency (ETOF, in openings per minute) for all 22 ears mean values ± standard deviation are given with 95% confidence intervals and median

ID	ETOP			ETOD			ETOF
	Mean ± std	95% CI	Median	Mean ± std	95% CI	Median	Median
1	38.3 ± 29.7	21.2–55.5	31.8	4.9 ± 3.3	2.9–6.8	4.6	3
2	31.2 ± 76.6	−8.2–70.6	12	3.3 ± 1.1	2.7–3.9	3.1	4.25
3	69.6 ± 17.3	57.2–82.0	64	3.2 ± 0.9	2.5–3.8	3.0	2.5
4	183.0 ± 23.8	145.2–220.8	184	2.5 ± 0.8	1.2–3.9	2.3	1
5	9.0 ± 6.7	6.3–11.7	8	1.4 ± 1.0	1.0–1.8	0.9	6.5
6	58.4 ± 35.9	32.7–84.1	47.5	2.3 ± 0.7	1.7–2.8	2.5	2.5
7	10.1 ± 5.7	8.3–12.0	12	1.8 ± 0.7	1.5–2.0	1.8	9.75
8	21.2 ± 6.0	18.8–23.7	20.5	2.4 ± 0.6	2.2–2.7	2.3	6.5
9	27.0 ± 10.6	21.7–32.3	27	3.1 ± 1.0	2.6–3.6	3.2	4.5
10	8.9 ± 2.5	8.4–9.5	9	0.6 ± 0.2	0.6–0.6	0.6	17.5
11	20.8 ± 6.0	18.4–23.2	22	2.7 ± 1.1	2.3–3.1	2.7	6.8
Total	22.7 ± 33.7	18.6–26.8	13	2.0 ± 1.6	1.8–2.2	1.8	4.5

### Comparison with Valsalva and Toynbee maneuvers

Out of all participants, six [6] chose the Valsalva as their preferred equalization technique during the second pressure chamber measurement. ETOP for all 12 ears was 17 ± 18.4 mbar (range 2–128 mbar, 95% CI 8.3–63.3) with a median of 12.5 mbar. ETOD for Valsalva was 2.2 ± 2.1 s (range 0.4–10.4 s, 95% CI 1.8–2.5) with a median of 1.4 s. ETOF was 6.8 ± 5 (range 0–14.5, 95% CI 3.6–9.9) openings per minute with a median of 5.5 times.

Comparison between Valsalva and Frenzel within the group of six people showed a significantly higher ETOP (Wilcoxon signed rank test, *p* < 0.001) and longer ETOD (Wilcoxon signed rank test, *p* < 0.001) for the Valsalva maneuver. For ETOF, no significant difference was found between the Valsalva and Frenzel maneuver.

In total, five [5] participants chose Toynbee as equalization maneuver during the second pressure chamber measurement. ETOP for the Toynbee maneuver was 15.1 ± 26.3 mbar (range 1–194, 95% CI 11.4–18.9) with a median of 10 mbar. ETOD was 0.8 ± 0.5 s (range 0.2–2.6, 95% CI 0.7–0.8) with a median of 0.6 s and ETOF was 9.5 ± 7.3 openings per minute (range 1–19.5, 95% CI 4.3–14.6) with a median of 11.8 openings per minute.

**Fig. 4:**
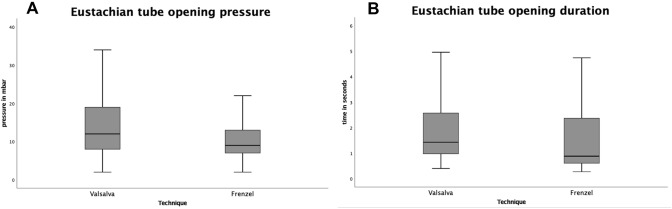
Comparison of Eustachian tube opening pressure and duration between the Frenzel and Valsalva maneuver Eustachian tube opening pressure (**A**) in mbar and duration (**B**) in seconds during the Frenzel (*n* = 12) and Valsalva (*n* = 12) maneuver. Wilcoxon signed rank test showed a higher opening pressure (*p* < 0.001) and longer opening duration (*p* < 0.001) for the Valsalva maneuver

**Fig. 5:**
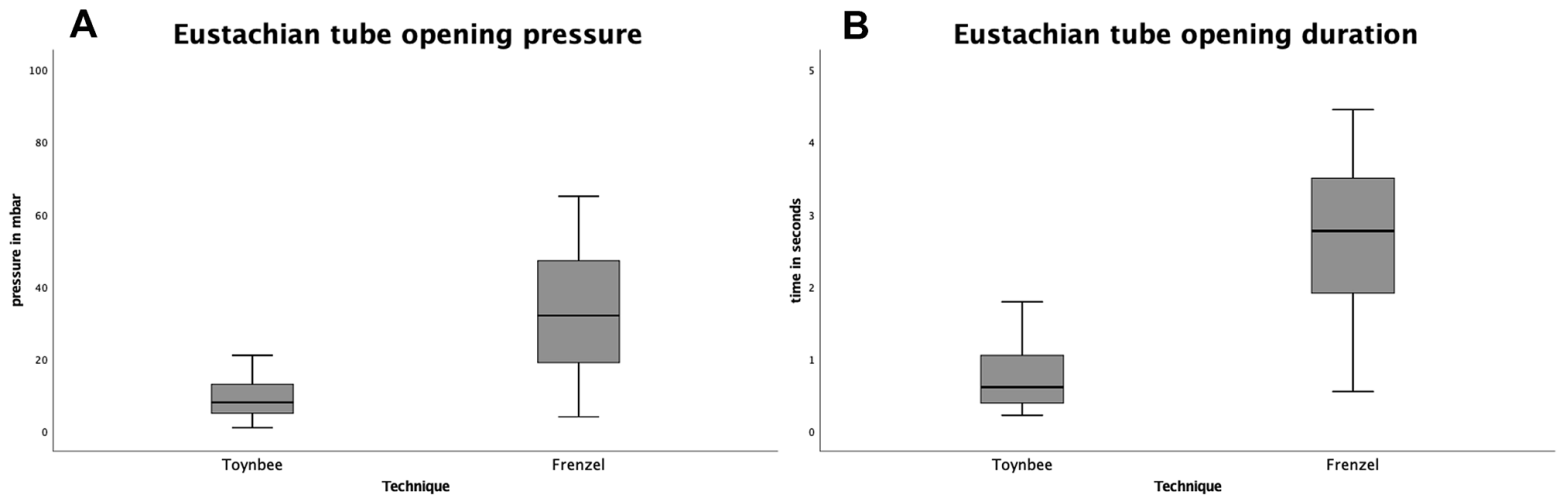
Comparison of Eustachian tube opening pressure and duration between the Frenzel and Toynbee maneuver Eustachian tube opening pressure (**A**) in mbar and duration (**B**) in seconds during the Frenzel (*n* = 10) and Toynbee (*n* = 10) maneuver. Wilcoxon signed rank test showed a lower opening pressure (*p* < 0.001) and shorter opening duration (*p* < 0.001) for Toynbee maneuver

Comparison between Frenzel and Toynbee within the group of five people showed a significantly lower ETOP (Wilcoxon signed rank test, *p* < 0.001) and shorter ETOD (Wilcoxon signed rank test, *p* < 0.001) for the Toynbee maneuver. For ETOF, no significant difference was found between the Valsalva and Frenzel maneuver (Figs. 4, 5).

## Discussion

In the present prospective trial, we were able to characterize the ET function in 11 freedivers (22 ears) during the Frenzel maneuver. We could demonstrate that performance of valid measurements of the Frenzel maneuver under standardized conditions in a hypo/hyperbaric pressure chamber was feasible. Additionally, we were able to compare ET function during the Frenzel maneuver to commonly used pressure equalization techniques such as the Valsalva and Toynbee maneuvers.

If we look at the technical execution of the maneuvers, we can see parallels and differences. As with the Valsalva maneuver, the Frenzel maneuver also triggers pressure in the nasopharynx that exceeds the ETOP. While the pressure in the Valsalva maneuver is increased by increasing the intrapulmonary pressure while having the mouth and nose closed at the same time, in the Frenzel maneuver, only the pressure in the nose and nasopharynx is increased by closing the soft palate. However, an over-pressure acts on the Eustachian tube in both maneuvers, resulting in Eustachian tube opening. It was assumed that both ETOP and ETOD should be similar when performing the Valsalva maneuver compared to the Frenzel. The Toynbee maneuver is performed without creating an over-pressure in the nasopharyngeal space and allows Eustachian tube opening through muscle activity. A shorter Eustachian tube opening and less possibility of pressure equalization by Toynbee in comparison to the Frenzel maneuver seems logical and was thus presumed. Fittingly, the pressure curve during the Frenzel maneuver is reminiscent of pressure curves during the Valsalva maneuver [8, 9]. However, the comparison of ETOP between Frenzel and Valsalva revealed a significantly higher ETOP for the Valsalva maneuver. Compared to the Toynbee maneuver, ETOP during Frenzel was significantly higher. In an earlier study by Mikolajczak et al., even higher standard ETOP values for the Valsalva and Toynbee maneuver were reported [8]. However, the latter values were measured on healthy non-diving participants. In the present trial, we focused on experienced freedivers, which might equalize pressure in a different way or have more experience with equalization. In our study, ETOD was shorter during the Frenzel maneuver than during the Valsalva but longer than during the Toynbee maneuver. Mikolajczak et al. reported a mean ET opening duration of 2.7 ± 1.9 of seconds for Valsalva and 0.8 ± 0.5 s for Toynbee [8] which is comparable to the demonstrated results of ETOD during the Valsalva and Frenzel maneuver (Table 1). The inter and intraindividual variability of values, which were also present in the current study, might explain the discrepancy in values. Bunne et al. also reported high intraindividual variability of values, measured on different test days [14]. Interestingly, the frequency of active pressure equalization was similar for all three techniques. Since each maneuver for active pressure equalization is activated individually, the actual ET opening frequency depends on the individual’s threshold, i.e. pain or pressure threshold. Therefore, ETOF might pose as the least objective parameter for ET function in the current study.

Otoscopic findings revealed more hyperaemia of the tympanic membrane (higher TEED level). However, the study design included that participants left the chamber for approximately 15 min between the measurements and the order of the different maneuvers was predetermined. Therefore, it remains unclear whether the difference in clinical otoscopic findings was caused by cumulative pressure exposure or by repeated pressure equalizing maneuvers.

Since no further investigations were conducted regarding the effectiveness of the Frenzel or Valsalva techniques, their clinical application also has to be taken into account. The majority of apnea divers preferably use Frenzel maneuver during deep dives, whereas scuba divers preferably equalize with Valsalva or Toynbee. The latter could of cause be explained by the lack of knowledge of the Frenzel maneuver. However, the wide application of the maneuver by experienced freedivers points to the superiority of the method. Compared to Valsalva, Frenzel can be performed hands-free with a nose clip. However, in the current study, it was performed using one hand. Furthermore, because the intrathoracic pressure does not increase during Frenzel, it does not influence cardiac hemodynamics as much as the Valsalva maneuver and diminishes the risk of the right-left shunt in the atrium [2]. In summary, the Frenzel maneuver was at least as effective as the Valsalva maneuver concerning ET openings in this study.

The discussion about the appropriate equalization technique and their appropriate application is still a hot topic among the professionals in apnea and scuba diving. In various prospective studies in scuba divers, inappropriate use of Valsalva was shown to be associated with a higher number of pressure related traumas of the middle ear (i.e. barotrauma) [12, 15, 16]. Therefore, less forceful and more frequent equalization is often recommended to novices in scuba diving. However, because of variable anatomy of the middle ear, there is no standard technique for pressure equalization. Furthermore, no single technique is considered the safest or most appropriate for equalizing middle ear pressure. When one system fails, the use of alternative approaches will improve success [7]. Therefore, various techniques should be taught during diving education. To our knowledge, only few scuba divers and instructors are familiar with the Frenzel technique. Its inclusion into the standard educational diving program might facilitate management of pressure equalization problems for students.

The present study is the first prospective observational trial and only includes 11 participants (22 ears). Due to this relatively low number, generalization of the results is difficult. With regard to the subgroups of divers that performed the Valsalva and Toynbee, the number and thus the reliability of the results is again limited. As already observed in previous studies, the ET function values undergo high individual variations [2, 8, 9]. Small study groups do not allow a meaningful randomization. For this reason, participants were free to choose their pressure equalization technique. In future studies, it would be desirable to change the sequence of the pressure equalization techniques and to perform decompression maneuvers with the same ETOF without compression or decompression to make a statement about clinically significant otoscopic findings. However, despite the small number and individual variations, we were able to demonstrate stable results in our study with a prospective design.

In this first prospective observational trial, we could establish values for ET function during the Frenzel maneuver in a standardized environment in a hypo/hyperbaric pressure chamber and compare them to the ET function parameters during the Valsalva and Toynbee maneuvers. The Frenzel maneuver was shown to be at least as effective as the Valsalva maneuver concerning ET opening. We believe that knowledge of the Frenzel technique might facilitate the pressure equalization during diving and recommend implementation of an appropriate equalization training in apnea and scuba diving education.

## Supplementary Information

Below is the link to the electronic supplementary material.

Supplementary file1 (PDF 840 KB)

## Data Availability

Source data is available by the author upon request.
