# Supplementary figures and images for: Prospective study on the Eustachian tube function during Frenzel maneuver in a hypobaric/hyperbaric pressure chamber
Source: Eur Arch Otorhinolaryngol. 2021 Jun 6;279(4):1843–50. doi: 10.1007/s00405-021-06888-1 (PMC8930945; doi:10.1007/s00405-021-06888-1)

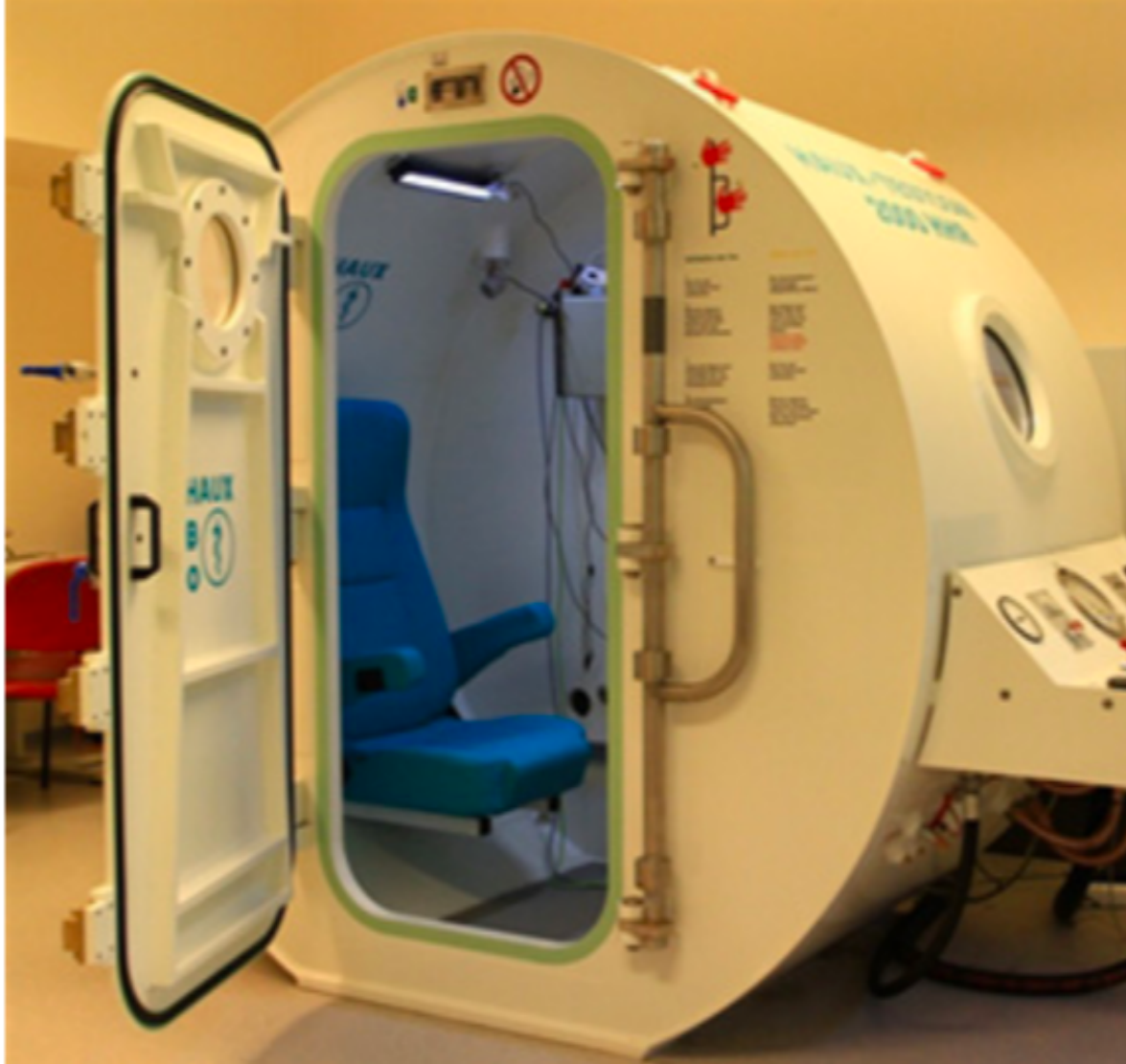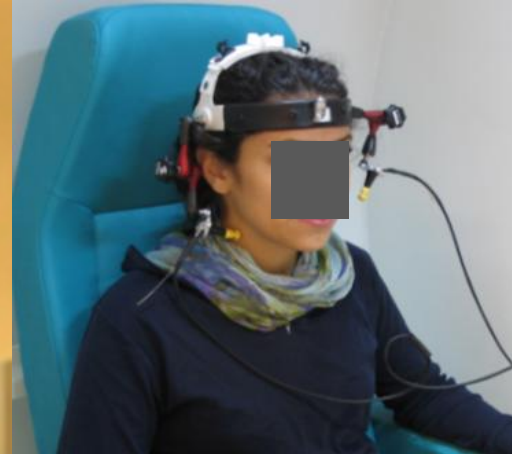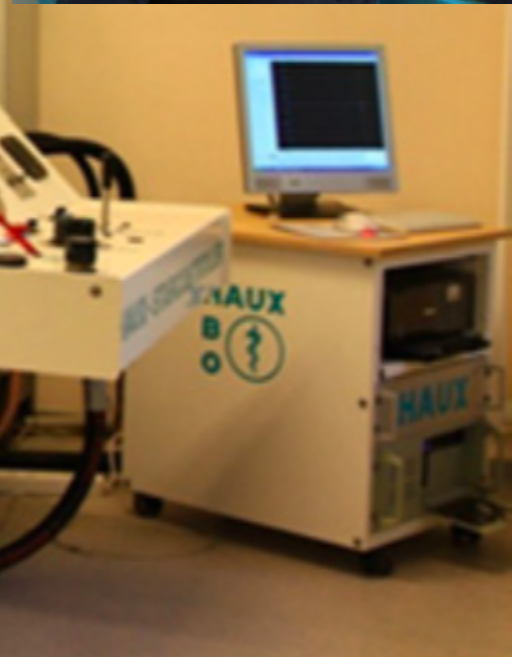

Supplement: Supplementary file 1 — Supplementary file1 (PDF 840 KB) [file 405_2021_6888_MOESM1_ESM.pdf]
